# Mycelial mass production of fungi *Duddingtonia flagrans* and *Monacrosporium thaumasium* under different culture conditions

**DOI:** 10.1186/1756-0500-6-340

**Published:** 2013-08-29

**Authors:** Manoel Eduardo da Silva, Jackson Victor de Araújo, Fabio Ribeiro Braga, Luana Alcântara Borges, Filippe Elias F Soares, Walter dos Santos Lima, Marcos Pezzi Guimarães

**Affiliations:** 1 Departamento de Veterinária, UFV, Viçosa, MG, Brasil; 2 URECO/EPAMIG, Pitangui, MG, Brasil; 3 Departamento de Bioquímica e Biologia Molecular, UFV, Viçosa, MG, Brasil; 4 Universidade Vila Velha- UVV, Vila Velha, ES, Brasil; 5 Instituto de Ciências Biológicas, UFMG, Belo Horizonte, MG, Brasil

**Keywords:** Nematophagous fungi, *Duddingtonia flagrans*, *Monacrosporium thaumasium*, Biological control, Fungal mycelium

## Abstract

**Background:**

*Duddingtonia flagrans* and *Monacrosporium thaumasium* are promising fungus species in veterinary biological control of gastrointestinal nematodes because of their production capacity of fungal structures (conidia and/or chlamydospores), growth efficiency in laboratory solid media and especially their predatory capacity. However, their large-scale production remains a challenge. This work aimed at evaluating the mycelial mass production of *D*. *flagrans* (AC001 and CG722) and *M*. *thaumasium* (NF34A) nematophagous fungi under different culture conditions.

**Results:**

The results did not present significant differences (p > 0.05) in mycelia mass production between the isolates cultured under pH 4.0. Furthermore, after 168 hrs., the isolate CG722 presented a lower production of mycelial mass in medium CM (corn meal) (p < 0.05).

**Conclusion:**

We therefore concluded the use of culture media SD (soy dextrose) and CG (corn grits) at pH values between 6.0 and 7.0 is suitable for high mycelial mass production of *D*. *flagrans* and *M*. *thaumasium*.

## Background

*Duddingtonia flagrans* and *Monacrosporium thaumasium* are promising fungus species in veterinary biological control of gastrointestinal nematodes because of their production capacity of fungal structures (conidia and/or chlamydospores), growth efficiency in laboratory solid media and especially their predatory capacity [1]. However, the application of these organisms as biological control agents [2,3] is still facing major challenges in large-scale production and storage. After isolation, these organisms need to be preserved for long periods of time and evaluated in order to be selected as potentially useful in control programs. Their maintenance in the laboratory is a basic requisite for successful biological control programs, though technical difficulties have impaired this. The fungi maintenance process requires periodic replication, exposure of the culture to contaminations and favouring mutations that can alter their predatory capacity [2,4,5].

Biological control with predatory nematophagous fungi is a viable option [1]. One of the principal advantages is its ability to survive for long periods under laboratory conditions; however, some isolates may lose their predatory activity [2].

According to Nozaki *et al*. [6], the conditions for fungus growth are not always the same for sporulation. It is also known that some culture media are favoured more than others for the sporulation of fungi. Economically viable methods for the development of fungal material production in the laboratory are necessary and an important step in enabling the commercial production of nematophagous fungi [1]. This work aimed at evaluating the mycelia mass production of *D*. *flagrans* (AC001 and CG722) and *M*. *thaumasium* (NF34A) nematophagous fungi under different culture media and pHs and after different incubation times.

## Methods

### Fungi

Two isolates of the fungus *Duddingtonia flagrans* (AC001 and CG722) and one of *Monacrosporium thaumasium* (NF34A) which originated from Brazilian soil, were maintained by continuous transfer to solid media culture in the Laboratory of Parasitology of the Department of Veterinary of the Federal University of Viçosa. Specimens were kept at 4°C in 2% corn meal agar (2% CMA) and protected from light.

### Mycelia mass production

Five fragments with approximately 4 mm of each isolate (AC001, CG722 and NF34A) previously cultured in 2%CMA were transferred to Erlenmeyer flasks containing 50 ml of the liquid media:

(1) Soy dextrose (SD): 200 g of cooked grain for 30 min in 1 l of water, 40 g of dextrose added.

(2) Potato dextrose (PD): 200 g of cooked and peeled potatoes for 30 min in 1 l of water, 40 g of dextrose added.

(3) Corn grits (CG): 200 g of cooked corn grits for 30 min in 1 l of water, 40 g of dextrose added.

(4) Corn meal agar (CM): 17 g of corn extract (DIFCO) diluted in 1 l of boiling water (100°C). The agar was removed from the medium through filtration using a Tamis filter 106.

The soy grain [*Glycine max* (L.) Merr.] contained 39.01% of brute protein, 22.80% of oleic fatty acids and 1.91% of alanine amino acids. The corn grain (*Zea mays*) contained 9.11% of brute protein, 28.45% of oleic acid fatty acids and 0.66% alanine amino acids. The potato (*Solanum tuberosum* L.) contained 2.39% brute protein [7]. These nutrients, according to Rosenweig [8] and Dijksterhuis et al. [9], are considered essential for fungal growth and sporulation.

### pH of culture media

The described media above (SD, PD, CG and CM) had their pH measured by a pH meter and were adjusted to 4.0, 5.0, 6.0 and 7.0 through the addition of NaOH (1 N) or HCl (1 N) diluted in distilled water.

### Incubation times

Each fungal isolate for all culture media and pHs were incubated at 26.5°C under constant agitation (65 rpm). After growth, three samples of each isolate from the different culture conditions were collected after 24, 48, 72, 96 and 168 h of incubation. Samples were filtrated with ME 24 0.2 μm filter in Kitazato flasks and using a vacuum pump. Filtrated samples were maintained in BOD and incubated at 50°C and weighed daily.

### Statistical analysis

Data obtained from the mycelial mass growth of the tested fungi (AC001, NF34A and CG722) from the different culture media, times and pHs were subjected to analysis of variance (ANOVA). Subsequently, the efficiency of the mycelial growth in the different culture media, times, and pH was evaluated by Tukey’s test at 1% probability.

## Results

The results for mycelial mass production of the three tested fungal isolates (AC001, CG722 and NF34A) at different pH values and times of cultivation are shown in Table 1.

**Table 1:** **Mycelial mass production of the fungi *D***. ***flagrans *and *M***. ***thaumasium *under different culture conditions**

**Times (hs)**	**Isolates**	**pH 5**		**pH 6**		**pH 7**
		**SD**	**CG**	**SD**	**CG**	**CG**
24	AC001	0.40 ± 0.12A	1.40 ± 0.25A	0.16 ± 0.08A	1.20 ± 0.88A	1.86 ± 0.42A
	NF34A	0.37 ± 0.01A	0.36 ± 0.03A	0.34 ± 0.04A	0.32 ± 0.08A	0.35 ± 0.05AB
	CG722	0.22 ± 0.05A	0.40 ± 0.23A	0.15 ± 0.02A	0.61 ± 0.25A	0.14 ± 0.20A
48	AC001	0.59 ± 0.08B	1.44 ± 0.16A	0.50 ± 0.12A	1.63 ± 0.51A	1.11 ± 0.58AB
	NF34A	0.38 ± 0.04B	0.61 ± 0.06A	0.43 ± 0.10B	0.34 ± 0.02A	1.12 ± 0.60AB
	CG722	0.25 ± 0.07A	0.51 ± 0.30B	0.15 ± 0.01A	0.28 ± 0.25AB	0.40 ± 0.07B
72	AC001	0.64 ± 0.22A	1.68 ± 0.23B	0.73 ± 0.23A	1.30 ± 0.38AB	1.90 ± 0.40B
	NF34A	0.42 ± 0.01A	0.38 ± 0.42B	0.46 ± 0.05A	0.37 ± 0.04AB	0.37 ± 0.03B
	CG722	0.25 ± 0.18A	0.36 ± 0.58B	0.20 ± 0.04A	0.48 ± 0.24AB	0.45 ± 0.10B
96	AC001	1.10 ± 0.47A	1.14 ± 0.43B	0.59 ± 0.04A	1.06 ± 0.26AB	0.93 ± 0.16B
	NF34A	0.35 ± 0.02A	0.37 ± 0.02B	0.57 ± 0.09A	0.34 ± 0.03AB	0.37 ± 0.08B
	CG722	0.30 ± 0.02A	0.60 ± 0.12B	0.39 ± 0.05A	0.38 ± 0.19AB	0.34 ± 0.09B
168	AC001	1.17 ± 0.28A	1.02 ± 1.04B	1.15 ± 0.10A	1.50 ± 0.27AB	0.30 ± 0.86B
	NF34A	0.64 ± 0.16A	0.80 ± 0.16B	0.59 ± 0.11A	0.36 ± 0.04AB	0.38 ± 0.06B
	CG722	0.97 ± 0.28A	0.23 ± 0.05B	1.07 ± 0.50B	0.17 ± 0.03AB	0.14 ± 0.31B

Means and standard deviation expressed in grams.

Different capital letters in columns show statistical difference (p < 0.05 or p <0.01) by Tukey test.

In the present study, no differences were observed in mycelial mass (p > 0.05) between the tested fungal isolates (AC001, CG722 and NF34A) in any of the culture media at pH 4.0. For this pH value, there was also no influence observed for the different for the mycelial mass production.

On the other hand, we observed that the isolates AC001 and NF34A present different mycelial mass production in the SD culture medium at pH 5.0 after 48 hours of incubation compared to other incubation times and isolates (p <0.05). For the CG culture medium with this same pH value, the three tested isolates (AC001, and CG722 NF34A) showed no difference after 24 and 48 hours for the mycelial mass production. However, a difference was observed (p <0.01) for the mycelial mass production of the three tested fungal isolates after 72, 96 and 168 hours.

Regarding pH 6.0, a difference was observed (p <0.05) among the tested isolates in mycelial mass production for SD and CG culture media at the different times tested. Furthermore, in the SD culture medium, the isolate NF34A showed a higher mycelial mass after 48 hours of cultivation (p <0.01). However, the isolate CG722 showed higher mycelial mass after 168 hours of culture (p <0.01). For the CG culture medium, the three tested isolates (AC001, CG722 and NF34A) showed no difference after 24 and 48 hours. However, after 72, 96 and 168 hours, a difference was observed (p <0.01) with a tendency for higher mycelial mass for the higher periods of culture.

The isolates AC001, CG722 and NF34A present different growth rates at pH 7.0 after 24 and 48 hours of incubation (p <0.01). The growth at this pH also tends to be higher after longer incubation times.

## Discussion

Our results demonstrated that the nematophagous fungi *D*. *flagrans* and *M*. *thaumasium* can grow in culture using the media described, which are agro-industrial by-products, and present good mycelial mass production. Moreover, the optimisation of the culture time and culture medium pH can be important in the future application of these organisms as biological control agents and in their large-scale industrial production. According to Araújo and Ribeiro [10], the commercialisation of nematophagous fungi depends on their: [1] predatory efficiency, [2] stocking demands, [3] satisfactory growth rates in the laboratory and [4] acceptability of association to other isolates. Although predatory activity is not associated with mycelial growth, fungal growing (either chlamydospores or mycelia) is an important factor in terms of propagation and survival of these organisms in environmental conditions [11].

Although all the culture media used in our experiments have relatively low production costs, the culture time necessary for the mycelial mass production were different between the tested isolates of predatory fungi (AC001, NF34A and CG722). This result indicates possible saturation of the culture media, which depends on the inoculum, culture medium pH and medium type tested, thus corroborating other studies [1,12-15].

The highest mycelia production of the three fungal isolates maintained under the different culture media, culturing time and pH values (from 4 to 7), were achieved as follows: *D*. *flagrans* (AC001 isolate) growing in CG at pH 7, after 72 h incubation, resulted in 1.9 g mycelia production; *D*. *flagrans* (CG722 isolate) growing in SD at pH 6, after 168 h incubation time produced 1.07 g of mycelia and *M*. *thaumasium* (NF34A isolate) growing in CG at pH 7, after 48 h incubation produced 1.12 g mycelia mass. These results shown that neutral pH values resulted in a higher mycelial mass production. This result is in agreement with the works by Soprunov [16] and Mitsui [17] that observed a better growth of the nematophagous fungus *Arthrobotrys conoides* at a neutral pH. Dias and Ferraz [18] demonstrated that this fungus presented higher mycelial mass production in solid medium at six different pH values, ranging from 4.0 to 9.0. However, Gorlenko et al. [19] established pH values 6.0-7.0 as ideal for the mycelial mass development of *A*. *musiformis* and *A*. *conoides*. Therefore, nutritionally rich media at neutral pH values can be suggested as ideal for the development and production of fungi mycelial mass. This is particularly noticeable when associated with optimal temperature and culture times.

Of the culture media tested, soy dextrose (SD) and corn grits (CG) presented higher potential in mycelial mass production, possibly because of their bromatologic composition and nutritional enrichment essential for fungal growth, particularly the amino acid described by Rosenweig [8] and Dijksterhuis et al. [9] and their brute protein. These authors demonstrated higher nematophagous fungi growth in media containing oleic acid and D-alanine, carbon and energy sources of trap formation. Moreover, the media composition determines the quantity and quality of fungal mycelial growth and sporulation. In addition to the medium composition, others factors such as temperature and light are critical to the sporulation [20]. However, when a fungus grows better in a specific media, it is accepted that specific metabolites are involved [21]. Therefore, the methods of our work were similar to those described by Dias & Ferraz [18] who observed radial growth and fungal structures of *Arthrobotrys* spp in PDA (potato dextrose agar), BDA-P (potato dextrose agar; peptone, 10 g), YPSSA (yeast extract, 4 g; K_2_HPO_4_, 1 g; MgSO_4_.7H_2_O, 0.5 g; soluble starch, 20 g; agar, 20 g; distilled water, 1 l), CMA(corn meal agar) and corn meal-A (corn meal, 20 g; agar, 20 g; distilled water, 1 l) media. These authors also stated that the use of liquid media and mycelial mass weighing would be suitable techniques for the evaluation of mycelial growth. Field studies are necessary to evaluate the predatory ability of the tested fungal isolates (AC001, CG722 and NF34A) cultured at different pH values and culture times in order to prove their efficiency in biological control programs.

## Conclusion

The use of culture media SD (soy dextrose) and CG (corn grits) at pH values between 6.0 and 7.0 is suitable for high mycelial mass production of *D*. *flagrans* and *M. thaumasium*. More studies must be conducted to determine their nutritional demands.

The results of the present study will have an important implication in fungal production for further works focused to evaluate the oral administration of nematophagous fungal mycelia as a strategy of control of gastrointestinal parasitic nematodes of importance for livestock industry.

## Competing interests

The authors declare that they have no competing interests.

## Authors’ contributions

Silva, ME; Araújo, JV; Borges, LA; and Braga, FR contributed in the designing and coordinating the experiment and also in the preparation and revision of the manuscript. Silva, ME; Soares, FEF; Lima, WS and Guimarães, MP participated in the design of the study and performed the statistical analysis. All authors read and approved the final manuscript.

## Authors’ information

Jackson Victor de Araújo CNPq scholarship.
